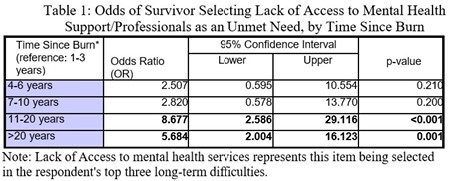# 66 Mental Health Support Is an Unmet Need for Long-Term Burn Survivors

**DOI:** 10.1093/jbcr/irae036.058

**Published:** 2024-04-17

**Authors:** Michael D Cobler-Lichter, Walter A Ramsey, Christopher F O'Neil, Mary Ishii, Shevonne S Satahoo, Joyce I Kaufman, Louis R Pizano, Tulay Koru-Sengul, Jose Szapocznik, Carl I Schulman

**Affiliations:** University of Miami, DeWitt Daughtry Family Department of Surgery - Division of Trauma, Acute Care Surgery, and Surgical Critical Care; Jackson Memorial Hospital - Ryder Trauma Center, Miami, Florida; Jackson Memorial Hospital, University of Miami, Miami, Florida; DeWitt Daughtry Family Department of Surgery, University of Miami, Miami, FL; Department of Public Health Sciences at University of Miami Miller School of Medicine, Miami, Florida; University of Miami Miller School of Medicine, Miami, Florida; University of Miami, DeWitt Daughtry Family Department of Surgery - Division of Trauma, Acute Care Surgery, and Surgical Critical Care; Jackson Memorial Hospital - Ryder Trauma Center, Miami, Florida; Jackson Memorial Hospital, University of Miami, Miami, Florida; DeWitt Daughtry Family Department of Surgery, University of Miami, Miami, FL; Department of Public Health Sciences at University of Miami Miller School of Medicine, Miami, Florida; University of Miami Miller School of Medicine, Miami, Florida; University of Miami, DeWitt Daughtry Family Department of Surgery - Division of Trauma, Acute Care Surgery, and Surgical Critical Care; Jackson Memorial Hospital - Ryder Trauma Center, Miami, Florida; Jackson Memorial Hospital, University of Miami, Miami, Florida; DeWitt Daughtry Family Department of Surgery, University of Miami, Miami, FL; Department of Public Health Sciences at University of Miami Miller School of Medicine, Miami, Florida; University of Miami Miller School of Medicine, Miami, Florida; University of Miami, DeWitt Daughtry Family Department of Surgery - Division of Trauma, Acute Care Surgery, and Surgical Critical Care; Jackson Memorial Hospital - Ryder Trauma Center, Miami, Florida; Jackson Memorial Hospital, University of Miami, Miami, Florida; DeWitt Daughtry Family Department of Surgery, University of Miami, Miami, FL; Department of Public Health Sciences at University of Miami Miller School of Medicine, Miami, Florida; University of Miami Miller School of Medicine, Miami, Florida; University of Miami, DeWitt Daughtry Family Department of Surgery - Division of Trauma, Acute Care Surgery, and Surgical Critical Care; Jackson Memorial Hospital - Ryder Trauma Center, Miami, Florida; Jackson Memorial Hospital, University of Miami, Miami, Florida; DeWitt Daughtry Family Department of Surgery, University of Miami, Miami, FL; Department of Public Health Sciences at University of Miami Miller School of Medicine, Miami, Florida; University of Miami Miller School of Medicine, Miami, Florida; University of Miami, DeWitt Daughtry Family Department of Surgery - Division of Trauma, Acute Care Surgery, and Surgical Critical Care; Jackson Memorial Hospital - Ryder Trauma Center, Miami, Florida; Jackson Memorial Hospital, University of Miami, Miami, Florida; DeWitt Daughtry Family Department of Surgery, University of Miami, Miami, FL; Department of Public Health Sciences at University of Miami Miller School of Medicine, Miami, Florida; University of Miami Miller School of Medicine, Miami, Florida; University of Miami, DeWitt Daughtry Family Department of Surgery - Division of Trauma, Acute Care Surgery, and Surgical Critical Care; Jackson Memorial Hospital - Ryder Trauma Center, Miami, Florida; Jackson Memorial Hospital, University of Miami, Miami, Florida; DeWitt Daughtry Family Department of Surgery, University of Miami, Miami, FL; Department of Public Health Sciences at University of Miami Miller School of Medicine, Miami, Florida; University of Miami Miller School of Medicine, Miami, Florida; University of Miami, DeWitt Daughtry Family Department of Surgery - Division of Trauma, Acute Care Surgery, and Surgical Critical Care; Jackson Memorial Hospital - Ryder Trauma Center, Miami, Florida; Jackson Memorial Hospital, University of Miami, Miami, Florida; DeWitt Daughtry Family Department of Surgery, University of Miami, Miami, FL; Department of Public Health Sciences at University of Miami Miller School of Medicine, Miami, Florida; University of Miami Miller School of Medicine, Miami, Florida; University of Miami, DeWitt Daughtry Family Department of Surgery - Division of Trauma, Acute Care Surgery, and Surgical Critical Care; Jackson Memorial Hospital - Ryder Trauma Center, Miami, Florida; Jackson Memorial Hospital, University of Miami, Miami, Florida; DeWitt Daughtry Family Department of Surgery, University of Miami, Miami, FL; Department of Public Health Sciences at University of Miami Miller School of Medicine, Miami, Florida; University of Miami Miller School of Medicine, Miami, Florida; University of Miami, DeWitt Daughtry Family Department of Surgery - Division of Trauma, Acute Care Surgery, and Surgical Critical Care; Jackson Memorial Hospital - Ryder Trauma Center, Miami, Florida; Jackson Memorial Hospital, University of Miami, Miami, Florida; DeWitt Daughtry Family Department of Surgery, University of Miami, Miami, FL; Department of Public Health Sciences at University of Miami Miller School of Medicine, Miami, Florida; University of Miami Miller School of Medicine, Miami, Florida

## Abstract

**Introduction:**

A National Trauma Research Action Plan identified the involvement of burn survivors as critical informants to determine the direction of research and clinical progress in the field. This study surveyed a nationwide sample of burn survivors to identify gaps in care and assess the importance of long-term challenges reported by survivors.

**Methods:**

We prospectively surveyed burn survivors ages ≥18 from around the United States from March-June 2023. Survivors were reached through social media and email contact with the Phoenix Society for Burn Survivors. We elicited demographic info, burn history, continued impact of various symptoms on quality of life, treatments used, long-term difficulties faced, unmet needs, and advice for other survivors. Statistical analysis was performed to test our hypothesis that lack of access to mental health support/professionals would be identified as an unmet need in long-term burn survivors.

**Results:**

Of 178 survey respondents (60% Female, 78% White) the majority were at least ten years removed from the date of their burn injury (n=94, 53%). Compared to those with less than 3 years from their burn injury date, individuals with greater than 10 years, were at least 5 times more likely to note lack of access to mental health support [11-20 years OR 8.7, p< 0.001; >20 years OR 5.7, p=0.001] (See Table 1). Of note, despite a small number of Spanish speaking respondents, 60% of Spanish speakers reported lack of access to support groups was among their greatest unmet needs, compared to 37% of English speakers (p=0.184).

**Conclusions:**

This study highlights the need for ongoing access to mental health resources throughout the entire lifespan of burn survivors. Our findings emphasize that burn injury is not just an acute ailment, but a complex condition that evolves into a chronic, multifaceted disease. Additional studies should focus on the experiences of Spanish-speaking burn survivors, as this group has a clinically meaningful lack of access to support groups and was poorly represented in our sample.

**Applicability of Research to Practice:**

This research calls for increased mental health support for long-term burn survivors. Providers must consider the chronic physical and psychological aspects of burn injury, striving to better equip patients for the future.